# State Pharmaceutical Association

**Published:** 1894-05

**Authors:** 


**State Pharmacal Association.**—At this writing, May 9th, the Texas State Pharmacal Association is in session in this city, with a fair attendance. Believing, with the doctors, who met here in convention week before last, that all work and no play makes Jack a dull boy, they have gone up the great lake on the Ben Hur this afternoon, and are having a good time. There are many distinguished druggists amongst them. We met Professor Jas. Kennedy, Professor of Pharmacy in the Texas Medical College and Dean of the School of Pharmacy, and Mr. Morrison, the pioneer druggist of the Geyser City—Waco. The Association will adjourn to-morrow, the 10th, to meet this time next year, in Galveston. Mr. Carlton, of Morley Bros., of Austin, is the President elect. Prof. Kennedy informs the JOURNAL that the Association will award a gold medal of $50 value to the first graduate of the Texas School of Pharmacy. Good.

